# ELISA-R: an R-based method for robust ELISA data analysis

**DOI:** 10.3389/fimmu.2024.1427526

**Published:** 2024-10-02

**Authors:** Taru S. Dutt, John S. Spencer, Burton R. Karger, Amy Fox, Andres Obregon-Henao, Brendan K. Podell, G. Brooke Anderson, Marcela Henao-Tamayo

**Affiliations:** ^1^ Department of Microbiology, Immunology, and Pathology, Colorado State University, Fort Collins, CO, United States; ^2^ Mycobacterial Research Laboratories, Colorado State University, Fort Collins, CO, United States; ^3^ College of Osteopathic Medicine, University of New England, Biddeford, ME, United States; ^4^ Environmental and Radiological Health Sciences, Colorado State University, Fort Collins, CO, United States

**Keywords:** ELISA, *Mycobacterium tuberculosis*, *Mycobacterium leprae*, antibodies, data analysis, curve-fitting, endpoint titer

## Abstract

Enzyme-linked immunosorbent assay (ELISA) is a technique to detect the presence of an antigen or antibody in a sample. ELISA is a simple and cost-effective method that has been used for evaluating vaccine efficacy by detecting the presence of antibodies against viral/bacterial antigens and diagnosis of disease stages. Traditional ELISA data analysis utilizes a standard curve of known analyte, and the concentration of the unknown sample is determined by comparing its observed optical density against the standard curve. However, in the case of vaccine research for complicated bacteria such as *Mycobacterium tuberculosis* (Mtb), there is no prior information regarding the antigen against which high-affinity antibodies are generated and therefore plotting a standard curve is not feasible. Consequently, the analysis of ELISA data in this instance is based on a comparison between vaccinated and unvaccinated groups. However, to the best of our knowledge, no robust data analysis method exists for “non-standard curve” ELISA. In this paper, we provide a straightforward R-based ELISA data analysis method with open access that incorporates end-point titer determination and curve-fitting models. Our modified method allows for direct measurement data input from the instrument, cleaning and arranging the dataset in the required format, and preparing the final report with calculations while leaving the raw data file unchanged. As an illustration of our method, we provide an example from our published data in which we successfully used our method to compare anti-Mtb antibodies in vaccinated vs non-vaccinated mice.

## 1 Introduction

Enzyme-linked Immunosorbent assay (ELISA) has been widely used over the years as a serodiagnostic tool in infectious diseases, including tuberculosis (TB) and leprosy (1–12). After getting infected with a pathogen, our immune system starts producing antibodies to fight the infection. ELISA has been used to detect the presence of these antibodies for either diagnostic purposes or for evaluating vaccine efficacy (13). When assessing vaccine efficacy, ELISA can provide us with precise information regarding whether the candidate vaccine is inducing potent antibody responses, the number of antibodies produced in vaccinated and non-vaccinated groups, the specific antigen targeted by the antibodies, and the type of antibody isotype generated. ELISA is a cost-effective and efficient method for the initial evaluation of vaccine effectiveness, particularly in human subjects (14, 15).

TB, caused by *Mycobacterium tuberculosis* (Mtb), continues to be a significant global health challenge. The current vaccine, Bacille Calmette-Guerin (BCG), shows variable efficacy, necessitating better vaccination strategies (16, 17). ELISA is a powerful tool for measuring immune responses, particularly antibodies or cytokines in the blood, which are essential for determining the efficacy of a vaccine or comprehending the various stages of TB (9, 18–20). In our study, we explored how mucosal exposure to non-tuberculous mycobacterium (NTM) affects B cell-mediated immunity and the efficacy of BCG vaccination against pulmonary TB, leveraging ELISA to quantify these immune responses (21). NTMs are mycobacterium that do not cause TB but are ubiquitous in the environment (22–26). Therefore, most populations in the low-middle income countries and even high-income countries are exposed to NTMs almost daily. However, how continuous NTM exposure affect the immune response to Mtb is largely unknown. Previous research has highlighted the potential of NTMs to modulate immune responses to TB due to shared antigens with Mtb and BCG (27–30). Studies using ELISA have shown mixed results: some report that NTM exposure can mask BCG-induced protection, while others suggest enhanced protection. While the role of NTMs in modulating BCG efficacy has been studied, the exact mechanisms, remain unclear. Therefore, there is a need for comprehensive studies. Here we used ELISA to measure the specific antibody responses induced by NTMs against Mtb. In our study, we investigated how mucosal exposure to NTMs affects B cell-mediated immunity and enhances the protective efficacy of BCG vaccination against pulmonary TB. We hypothesized that NTM exposure increases B cell influx and anti-Mtb antibody production in the lungs (due to ~85% genetic similarity between NTM and Mtb), leading to reduced bacterial burden and improved protection, as measured by ELISA. This study developed a mouse model mimicking human BCG vaccination followed by continuous NTM exposure via drinking water. Mice were divided into four groups: BCG only, NTM only, BCG plus low-dose NTM, and BCG plus high-dose NTM. After BCG vaccination and NTM exposure, mice were challenged with Mtb. ELISA was used to quantify anti-Mtb IgG and IgA antibodies in serum and bronchoalveolar lavage fluid (BALF). This data has been employed to develop our ELISA-R method and to quantify the anti-Mtb immune response in mice that were exposed to NTM versus those that were not.

**Figure 1:**
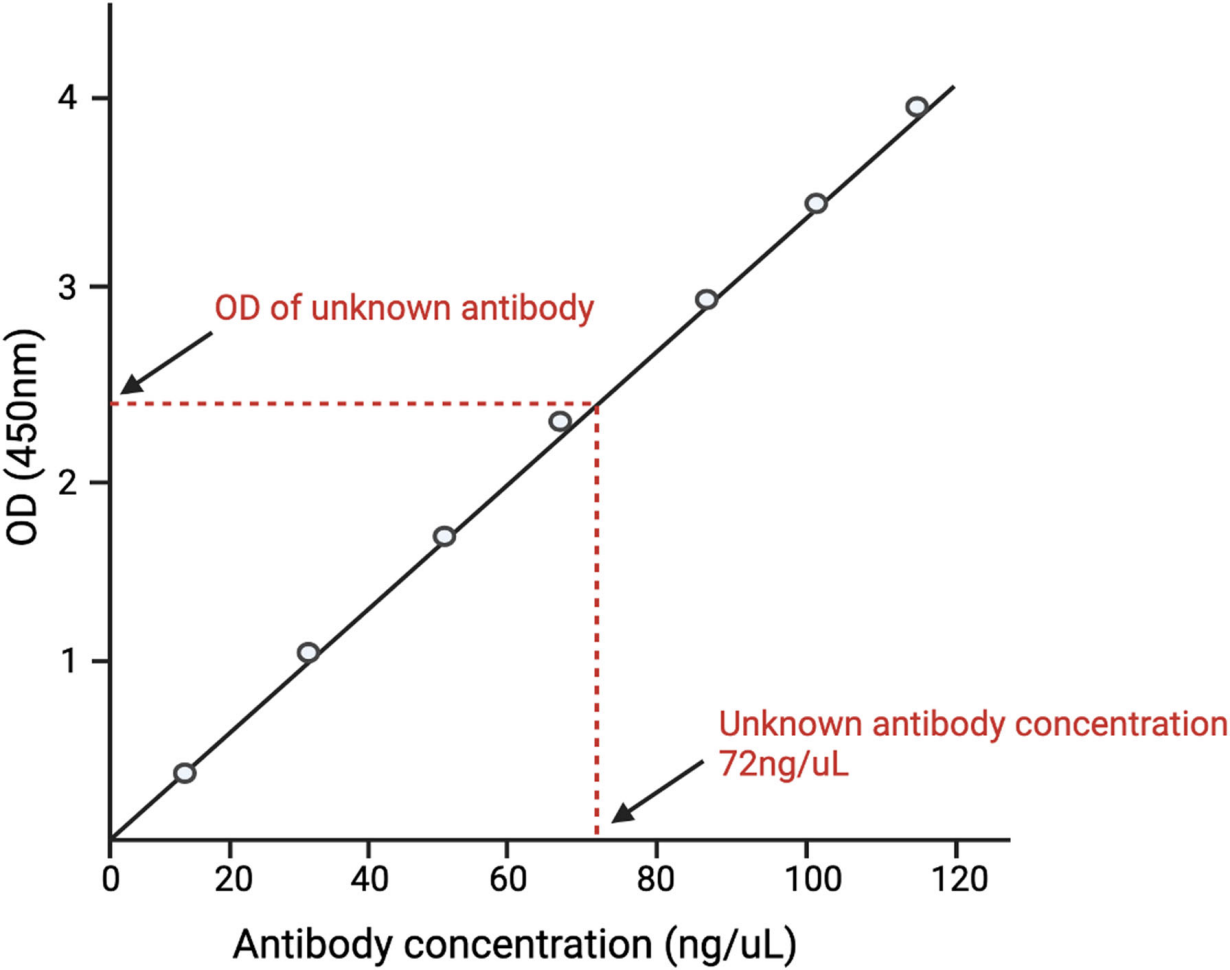
A depiction of the standard curve method of analyzing ELISA data. A standard curve is generated by plotting different concentrations of known antigens against the optical density (O.D.) of each concentration. The O.D. of the unknown antigen is then measured. The concentration of the unknown antigen is then determined by matching the O.D. of the unknown antigen with the O.D. of the known antigen and finding its respective concentration.

ELISA data analysis is as critical as conducting the assay itself. The correct data analysis ensures the accuracy, reliability, and interpretability of results. In the case of well-characterized viruses or known analytes, data analysis is generally uncomplicated and adheres to the standard curve method (31). In the standard curve method, the known antigen or antibody is plotted against varying concentrations of known antibody/antigen. The concentration of the unknown analyte is subsequently determined by comparing the observed value of the unknown analyte against this standard curve (**Figure 1**).

On the contrary, vaccine research, particularly that involving complex microorganisms like Mtb having 4000 genes coding for ~4000 proteins, typically entails examining the existence of antibodies directed against unidentified vaccine antigens. Consequently, the generation of a standard curve is not possible due to the lack of information regarding the antigen and antibody type. Antibody quantification is accomplished for this type of ELISA through the utilization of serial dilution of the samples and a non-standard curve method. As a result, the analysis of ELISA data in this instance is based on a comparison between vaccinated and unvaccinated groups. However, to the best of our knowledge, no robust data analysis method exists for “non-standard curve” ELISA.

**Figure 2:**
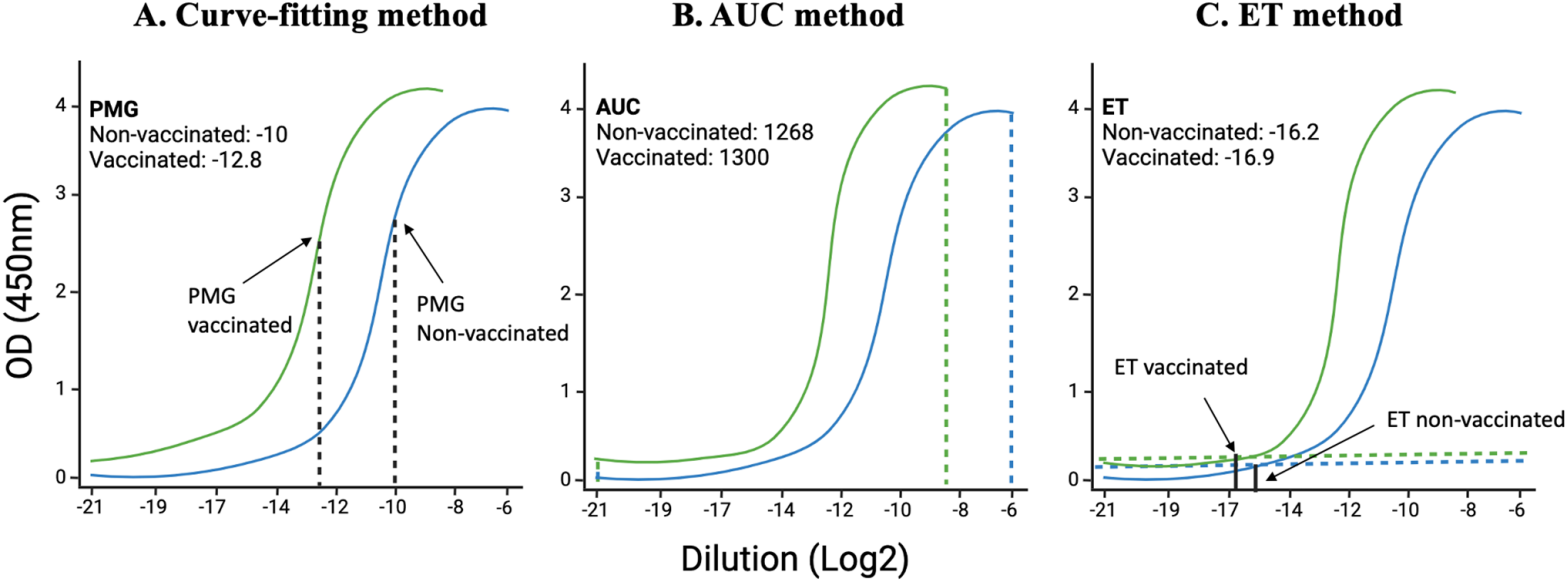
Illustration of curve-fitting, an area under the curve (AUC), and endpoint titer (ET) methods: Using data from Dutt et al., 2022, we have analyzed data using already established methods. **(A)** the previous curve-fitting method that calculates the point of maximum growth, **(B)** the AUC method that calculates the total area covered by each sample at different dilutions, and **(C)** the ET method that takes into consideration the least dilution at which the curve starts above the background.

The three common methods that have been used previously for ELISA data analysis without a standard curve are:

1. Fitting a sigmoid model curve: With this method, the sigmoidal curve is fit for each sample, and then estimated key parameters are compared to the points of maximum growth (PMG) (**Figure 2A**). When a sample has a higher concentration of protein to be measured, all dilutions of this sample have higher absorbance; the sigmoidal curve shifts towards a more diluted end (32, 33). While this method has high sensitivity and specificity, it requires complex algorithms and computational resources, needs sufficient data points for reliable curve fitting and low concentration samples may not yield accurate parameter estimates.
2. Area under the curve (AUC) method: AUC is defined as the definite integral between the two points in a curve (34–36). AUC for ELISA is calculated by estimating the parameters in a curve and then integrating the curve from the lowest concentration to the highest dilution (**Figure 2B**). AUC method offers the advantage of providing a comprehensive measure of the overall antibody response across all dilutions, making it useful for assessing the total immunogenicity in a sample. Its calculation is relatively simple compared to fitting a sigmoidal curve, involving straightforward integration of the absorbance data, which reduces computational demands. However, the AUC method has significant drawbacks. It cannot determine the endpoint titer, the maximum dilution at which the antibody remains detectable, thereby missing critical quantitative information. Additionally, the accuracy of the AUC is highly dependent on the correct estimation of curve parameters, which can be influenced by noise and variability in the data, potentially leading to less reliable results.
3. Endpoint titer (ET) method: The ET method in ELISA relies on selecting a cutoff absorbance level above the noise background (**Figure 2C**) (37, 38). The ET method in ELISA is advantageous due to its simplicity and ease of implementation. It quickly identifies the presence of antibodies by determining the highest dilution at which the antibody is still detectable, making it suitable for high-throughput screening. This method does not require complex computational tools or advanced expertise, allowing for rapid and straightforward analysis. However, the ET method produces discrete, less precise data, as dilutions are limited to specific values, potentially overlooking subtle differences in antibody concentrations. Additionally, the method is susceptible to background variability across different samples and wells, which can introduce bias and affect the accuracy of the cutoff determination. The lack of detailed quantitative information about antibody concentrations further limits its utility in comprehensive immunological studies.

Here, we have developed a comprehensive R-based ELISA method (ELISA-R) by integrating the fitting of a sigmoid model with the endpoint titer method to leverage the strengths of both approaches. The sigmoid model fitting provides high sensitivity and specificity by accurately modeling the concentration-absorbance relationship across a range of dilutions, allowing for precise quantification of protein levels. Meanwhile, the endpoint titer method offers simplicity and rapid identification of the highest dilution at which antibodies are still detectable, providing a clear and easily interpretable measure. Our ELISA-R method begins with the direct input of measurement data (O.D. values and sample information) from the instrument, ensuring a seamless transition from raw data to analysis. The data is then cleaned and arranged into the “tidy” format, preparing it for subsequent analysis steps. The sigmoid model fitting is applied to generate a detailed absorbance-concentration curve, extracting key parameters that represent the sample’s response. Simultaneously, the endpoint titer is determined by identifying the dilution level where the absorbance exceeds a predefined cutoff, indicating the presence of antibodies.

By combining these two methods, ELISA-R generates a single, specific value for each sample that reflects both the detailed quantitative information from the curve fitting and the practical, easily comparable endpoint titer. This dual approach enhances the robustness and reliability of the analysis, allowing for meaningful comparisons across different samples and groups. Additionally, ELISA-R ensures that the raw data file remains unchanged, preserving data integrity while producing a comprehensive final report with all necessary calculations and visualizations. This integrated method offers an accessible, efficient, and reproducible solution for ELISA data analysis, supporting a wide range of immunological research applications.

## 2 Methods

### 2.1 Data used for ELISA-R data analysis method development

For the ELISA-R development, we used our published data from Cell Reports (21). In this data, ELISA was performed to evaluate the presence of anti-Mtb IgA antibodies in the bronchoalveolar fluid (BALF) and serum of BCG vaccinated and BCG vaccinated-and non-tuberculous mycobacteria (NTM) exposed mice at 120 days post-Mtb infection. A detailed ELISA method has been described in the paper. But briefly, high binding 96‐half‐well microplates (Corning Life Sciences, cat#3690) were coated with 100 ng of Mtb HN878 lysate (BEI, cat# NR-14824) prepared in PBS and incubated overnight at 4°C. The next day, plates were washed five times with 180 µL of wash buffer (PBS + 0.05% Tween‐20), and non‐specific interactions were blocked using 180 µL of blocking buffer (PBS + 0.05% Tween‐20 + 2% BSA + 2% normal goat serum [Jackson ImmunoResearch Inc., cat#005-000-121 West Grove, PA, USA]). After 2 hours, plates were washed, and different dilutions of serum and BAL prepared in blocking buffer (1:50, 1:100, 1:200, 1:400, 1:800, 1:1600, 1:3200, 1:6400, 1:12800, and 1:25600) were added to the wells and incubated for one hour. Plates were then washed and incubated for 1 hour with horseradish peroxidase (HRP)‐conjugated anti‐mouse IgA secondary antibodies (Southern Biotech, cat# 1040-05) prepared in the blocking buffer. The colorimetric substrate was developed with the addition of 100 µl of TMB substrate (Thermo Fisher Scientific, Rockford, cat# ENN301), and the reaction was stopped by adding 50 µl of 1 M sulphuric acid. Absorbance was measured at 450 nm using a BioTek Synergy 2 plate reader (BioTek Instruments Inc., Winooski, VT, USA).

### 2.2 Creating a function for curve fitting model

To develop a more robust way of ELISA data analysis, we created a curve-fitting model function using the *nlsLM* function of the *minpack.lm* R package (39). nlsLM function from the minpack.lm package, is a powerful technique for fitting non-linear models to data. This approach is especially advantageous for ELISA data analysis, where the relationship between dilution and absorbance often follows a non-linear pattern. The nlsLM function in R is used for non-linear least squares fitting, a process that minimizes the sum of the squares of the residuals (the differences between observed and predicted values). This function is an enhancement of the traditional Levenberg-Marquardt algorithm, designed for more robust performance, particularly with complex, non-linear data (27). In the early 1960s, the Levenberg-Marquardt algorithm was developed to solve nonlinear least squares problems. Least squares problems arise in the context of fitting a parameterized mathematical model to a set of data points by minimizing an objective expressed as the sum of the squares of the errors between the model function and a set of data points. By minimizing the sum of squared residuals, non-linear models fitted using nlsLM will provide a better fit to the data, leading to more accurate predictions and inferences.

Hence, we used the following equation for curve fitting sigmoid model:


(1) f(x)=(a−d)/(1+(log_dilution/c)^b)) + d

Where,

a = maximum absorbance;

d = minimum absorbance;

c = point of maximum growth; and

b = slope at c.

This method takes into account the minimum and maximum absorbance values, the point of maximum growth, and the slope of the curve. This comprehensive approach ensures that all critical aspects of the data are considered.

### 2.3 Applying the values obtained from the curve-fitting model function as an input for the endpoint titer function

The traditional ET method, despite its widespread use in vaccinology for measuring antibody levels, has notable limitations that restrict its comprehensiveness. The method’s reliance on twofold serial dilutions, while generally adequate for distinguishing markedly different responses, may lack sensitivity in detecting subtle yet significant variations in antibody levels. Furthermore, the reproducibility and standardization of ET can be problematic due to variability in protocols and interpretation across different laboratories. Therefore, we created the function for endpoint titer (ET) evaluation. Our ET function is not based on the traditional minimum background for each sample but rather takes into account the minimum and maximum absorbance of each sample, the shape of the curve, and the slope of the curve. The input values for these parameters are taken from the output of the curve-fitting model.


(2) $$ET=c*{\left(((a-d)/(0.2-d))-1\right)}^{\left(1/b\right)}$$

Traditional endpoint titer calculations often rely on a fixed threshold, which may not be optimal for all samples. By using the fitted parameters from the curve, this method provides a more accurate and sample-specific endpoint titer.

### 2.4 Statistical analysis

Statistical significance between the two groups was determined using unpaired t-tests (*p* < 0.05) using the *ggpubr* package. If there are more than two groups, then Analysis of Variance (ANOVA) was applied. All codes have been generated and plotted using R (Version 4.1).

## 3 Results

### 3.1 Create the curve-fitting model using the NTM dataset

**Figure 3:**
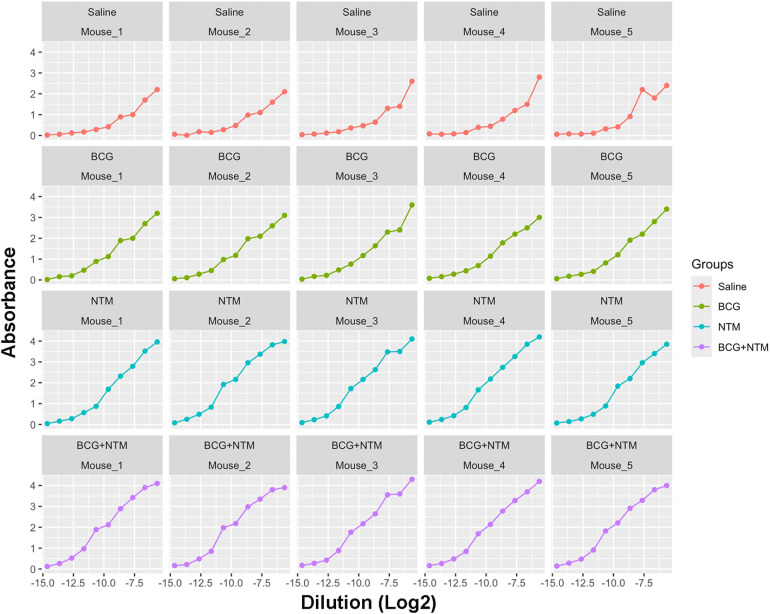
Curves generated for each mouse using a modified curve fitting model. After applying the curve-fitting model to our Dutt et al., 2022 dataset, our model generated the curves against dilution versus absorbance for each mouse. These curves then allow our modified curve fitting model to identify the average maximum absorbance, minimum absorbance, background, and slope of the curve based on each sample to enter into our method.

Using the NTM datasets, we first applied the approach of fitting a sigmoid model to each mouse in the Saline, BCG, NTM, and BCG+NTM groups. For this, we read the data obtained from Dutt et al., 2022 (21) (also provided in **Supplementary Table 1**) in R and reformatted the data to follow the principles of tidy data (40). We converted the dilutions to log base 2 to simplify the values and converted the data to a dataframe. We then applied the curve fitting model equation to each mouse in the group and evaluated the values for maximum absorbance, the slope at c, the point of maximum growth, and minimum absorbance (the values of the coefficients a, b, c, and d, respectively, in Equation 1. This function then results in a sigmoid curve of absorbance versus dilution (log2) for each mouse (**Figure 3**) and the values for a, b, c, and d for each mouse (**Supplementary Table 1**). We will then use the values of a, b, c, and d as input for the endpoint titer function.

### 3.2 Applying the curve-fitting model values to the endpoint titer function

**Figure 4:**
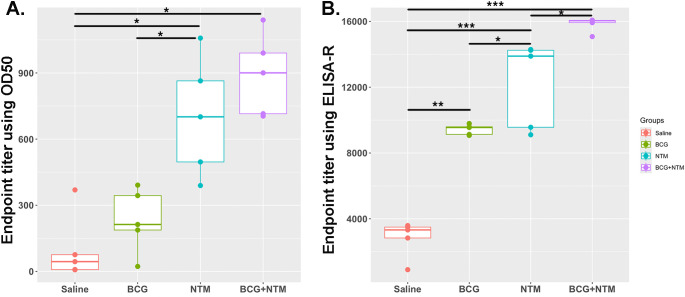
Comparative analysis of ELISA data analysis using traditional endpoint titer using O.D._50_ vs. endpoint titer calculated using ELISA-R. **(A)** Analysis of BCG-NTM mouse study ELISA data using traditional endpoint titer method that used O.D._50_ as a cut-off point. Data is plotted as the dilution of a sample that produces an O.D. value equal to 50% of the maximum O.D. observed for that sample. **(B)** Plot for Endpoint titer calculation using ELISA-R. In these graphs, the endpoint titer of IgA antibodies in the BALF of each mouse in the Saline, BCG, NTM, and BCG-NTM groups was compared with each other. We found that the BALF of BCG-NTM mice has a higher titer of IgA antibodies than the BALF of mice in Saline, BCG, or NTM groups. Statistical significance was calculated using ANOVA. *p<0.05, **p<0.005, ***p<0.0005.

Traditionally, ET has been calculated using O.D._50,_ which is the reciprocal of the highest sample dilution that gives a reading at O.D. of 50% of that sample. However, it does not take into account the minimum and maximum absorbance of each sample, the shape of the curve, and the slope of the curve. In our method, we are taking these important factors into account by combining the curve-fitting model with the ET function. We applied the values of a, b, c, and d to the above-mentioned ET equation and obtained the ET value of each mouse (**Supplementary Table 2**). We employed both the traditional Endpoint Titration (ET) method and the advanced ELISA-R method to analyze our data, as shown in **Figures 4A, B**, respectively. The comparison reveals significant differences between these two approaches. Using the traditional ET method, the endpoint titer values, analyzed with O.D._50_, ranged from 0 to 1200 dilutions. These values failed to accurately represent antibody strength and titer. Conversely, the ELISA-R method demonstrated superior performance by pinpointing the highest dilution at which the antibody effectively binds to the antigen. Furthermore, the ELISA-R method significantly reduced sample variability, providing more consistent and reliable results. This highlights ELISA-R’s ability to deliver precise and meaningful insights into antibody-antigen interactions, making it a more robust and dependable choice for ELISA data analysis.

### 3.3 Testing ELISA-R with clinical human serum samples

**Figure 5:**
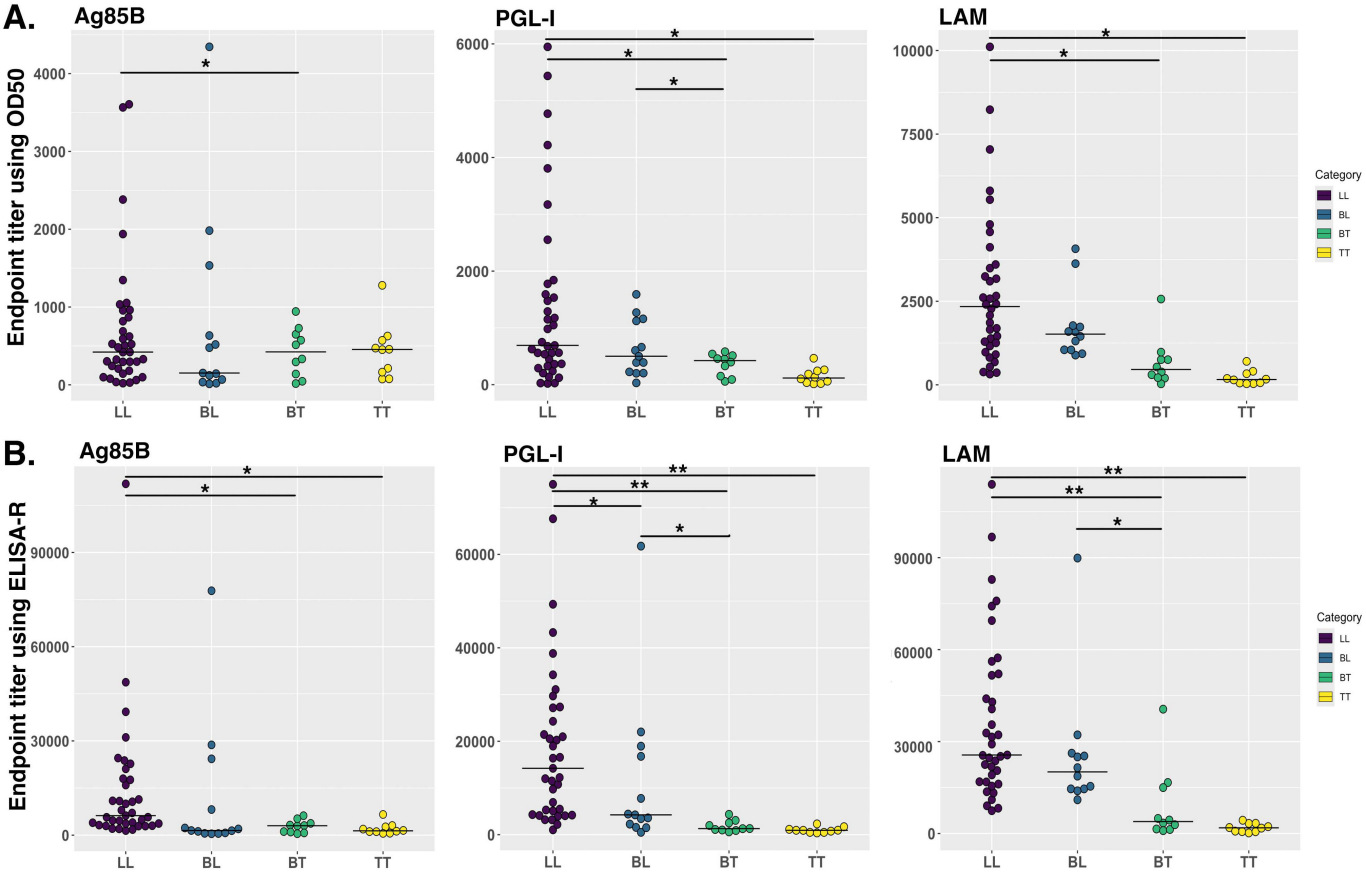
Comparison of human antibodies against *Mycobacterium leprae* antigens in different stages of leprosy patient cohorts. IgG antibodies against Ag85B and LAM and IgM antibodies against PGL-I were evaluated using indirect ELISA by Spencer et al. (41). We re-analyzed this data by using our ELISA-R for anti-Ag85B IgG, anti-PGL-I IgM and anti-LAM IgG in LL, BL, BT and TT patients. **(A)** Data was analyzed using the traditional ET method. **(B)** Data analyzed using ELISA-R. LL, polar lepromatous; BL, borderline lepromatous; BT, borderline tuberculoid; and TT, polar tuberculoid. Statistical significance has been calculated using ANOVA. *p<0.05, **p<0.005.

We developed ELISA-R using a dataset from a mouse model that is in a highly controlled environment with fewer variables. However, it is crucial to evaluate our method to other datasets, especially from human settings. Therefore, we have used the ELISA raw dataset with serial two-fold titration of the human serum from 1:100 to 1:102,400 with three antigens of *Mycobacterium leprae* (rAg85B, PGL-I, and LAM). There are 50 lepromatous patient sera, which are further divided into borderline lepromatous (BL, n = 13) or polar lepromatous (LL, n = 37), and 20 tuberculoid sera that are also divided into polar tuberculoid (TT, n = 10) and borderline tuberculoid (BT, n = 10). The information about these leprosy patient serum samples was previously published (41). The O.D. values for each dilution are plotted to go across each serum sample [**Supplementary Tables 3** (rAg85B), 4 (PGL-I), and **5** (LAM)]. We applied our ELISA-R method for the analysis of this dataset by taking into consideration each patient. We first read the data stored in Excel format in R, subtracted the baseline values of the patients from the respective wells, and arranged the data in a tidy format. We then applied ELISA-R to the data for antibodies against all three antigens, namely, rAg85B, PGL-I, and LAM. In addition, we applied the traditional ET method using O.D._50_ values to compare the difference between these two methods in human samples (with more variability). We found that anti-Ag85B, PGL-I, and LAM antibodies are highest in LL patients, followed by BL, BT, and TT, which were consistent with Spencer et al.’s published findings (**Figure 5**). In our analysis of human samples as well, the ELISA-R method consistently outperformed the traditional ET method, delivering cleaner, more accurate data with significantly reduced variability. This demonstrates ELISA-R’s superior reliability and precision in detecting and quantifying our target analytes.

## 4 Discussion

Quantifying and assessing the presence of antibodies directed against the bacterial or viral antigens that the vaccine targets is an essential element of vaccine research (42–44). However, quantifying antibodies and comparing them among different populations and vaccination groups can be challenging and time-consuming. Different methods are available to quantify antibodies and compare them among samples. However, all these methods have certain flaws that can misinterpret the data; for example, when we directly plot graphs based on optical density (O.D.), then we are not taking into consideration the baseline differences of each sample, the shape of the curve of the sample, and all the dilutions into consideration. Similarly, in the AUC method, there is a possibility that two or three samples can have similar AUC values. However, the antibody titers can be different. Therefore, the analysis of ELISA data should be more focused and consider all the crucial parameters into account, such as the background signal of each sample, O.D. values of samples at each dilution, and the slope of the sample curve.

Here, we have created ELISA-R, a robust R programming-based method for the analysis of ELISA data that takes into consideration all the above-mentioned challenges of ELISA data analysis. ELISA-R utilizes an advanced non-linear least squares curve-fitting model, enhanced with the Levenberg-Marquardt modification, to precisely identify critical parameters such as minimum and maximum absorption, background, and slope for each sample. These parameters are then used to compute accurate endpoint titer values, ensuring robust and reliable analysis. What sets ELISA-R apart is its exceptional time efficiency. ELISA-R seamlessly imports data directly from Excel, subtracts the blank of each sample, processes the data, and generates comprehensive graphs in under 15 minutes. This rapid processing time significantly enhances productivity, allowing researchers to focus on interpreting results rather than managing data. ELISA-R is designed with user-friendliness in mind, making it accessible to non-R programmers. We are also currently developing a graphical user interface to further simplify its use, ensuring that even those with minimal programming experience can easily navigate the software. Unlike traditional methods that consider only one or two parameters, ELISA-R takes into account five crucial parameters for each sample, resulting in a more robust and comprehensive analysis. Additionally, ELISA-R is free and open-source, making it an invaluable tool for researchers worldwide.

We developed ELISA-R using our published well-optimized ELISA data, as this data has been acquired using inbred mouse strain and, therefore, has less variability. Nonetheless, we have also applied our ELISA-R method to complicated datasets with larger variability, such as human patient data derived from different clinical forms of leprosy (**Figure 5**). ELISA-R worked successfully for both datasets and provided the same trend as we were getting by analyzing data manually (as confirmed by Dr. John Spencer, who analyzed the results manually). However, ELISA-R took significantly less time than the manual analysis. In addition, the results provided by our method are more robust as it takes different variables into account. All in all, ELISA-R is a robust and faster method of analyzing ELISA data, which is not limited to ELISA data alone but may be widely utilized for assays that involve optical density, dilutions, and curves, such as cytokine data.

### 4.1 Limitation of the ELISA-R

One significant challenge in ELISA data analysis is the potential for batch effects, which occur when assays for different groups of samples are conducted on different days, leading to variations in results due to technical inconsistencies rather than true biological differences. Factors such as changes in plate reader settings, the use of different plates, and other technical variations can introduce batch effects, complicating the analysis and interpretation of ELISA data. Currently, ELISA-R does not include methods to correct for batch effects and normalize the data, a limitation that impacts the accuracy and reliability of our analyses. To address this, we propose incorporating robust statistical methods for batch effect correction and data normalization in future versions of ELISA-R. One approach is to use mixed-effects models to account for both fixed effects (such as biological conditions) and random effects (such as batch variations), partitioning the variability attributable to batch differences and isolating the true biological signal. Another method is the empirical Bayes approach, such as the ComBat algorithm, which adjusts for batch effects by modeling batch-specific parameters and harmonizing the data across batches. Additionally, normalization techniques like quantile normalization can be used to make the distribution of absorbance values consistent across different batches, reducing the impact of batch-specific variations. By integrating these methods into ELISA-R, we aim to enhance its utility, making it a more powerful tool for ELISA data analysis, and ensuring that the results reflect true biological differences rather than technical artifacts. This will contribute to the reproducibility and reliability of ELISA experiments, leading to more accurate scientific discoveries.

### 4.2 Future prospective

We are currently developing a graphical user interface (GUI) for the ELISA-R method, utilizing the Shiny package in R. Our goal is to empower every researcher to analyze their ELISA data with greater accuracy and speed, all without the need for programming expertise. This user-friendly tool will make advanced ELISA data analysis accessible to all.

## Data Availability

The original contributions presented in the study are included in the article/**Supplementary Material**. Further inquiries can be directed to the corresponding authors.
